# Interspecific acoustic recognition in two European bat communities

**DOI:** 10.3389/fphys.2013.00192

**Published:** 2013-08-26

**Authors:** Adriana M. Dorado-Correa, Holger R. Goerlitz, Björn M. Siemers

**Affiliations:** ^1^Sensory Ecology Group, Max Planck Institute for OrnithologySeewiesen, Germany; ^2^Department of Biology II, Munich Graduate Program for Evolution, Ecology and Systematics, Ludwig-Maximilians-UniversityMunich, Germany

**Keywords:** acoustic communication, eavesdropping, echolocation, feeding buzz, interspecific communication, intraspecific communication, search calls

## Abstract

Echolocating bats emit echolocation calls for spatial orientation and foraging. These calls are often species-specific and are emitted at high intensity and repetition rate. Therefore, these calls could potentially function in intra- and/or inter-specific bat communication. For example, bats in the field approach playbacks of conspecific feeding buzzes, probably because feeding buzzes indicate an available foraging patch. In captivity, some species of bats recognize and distinguish the echolocation calls of different sympatric species. However, it is still unknown if and how acoustic species-recognition mediates interspecific interactions in the field. Here we aim to understand eavesdropping on bat echolocation calls within and across species boundaries in wild bats. We presented playbacks of conspecific and heterospecific search calls and feeding buzzes to four bat species with different foraging ecologies. The bats were generally more attracted by feeding buzzes than search calls and more by the calls of conspecifics than their heterospecifics. Furthermore, bats showed differential reaction to the calls of the heterospecifics. In particular, *Myotis capaccinii* reacted equally to the feeding buzzes of conspecifics and to ecologically more similar heterospecifics. Our results confirm eavesdropping on feeding buzzes at the intraspecific level in wild bats and provide the first experimental quantification of potential eavesdropping in European bats at the interspecific level. Our data support the hypothesis that bat echolocation calls have a communicative potential that allows interspecific, and potentially intraspecific, eavesdropping in the wild.

## Introduction

Many animals are able to recognize members of their own species (conspecifics) and/or to discriminate between members of their own and different species (heterospecific; Gerhardt and Huber, 2002). Some of them react with species-specific behavioral responses depending on the signal or cue of the heterospecific or conspecific (Seyfarth et al., 1980; Manser, 2001; Schuchmann and Siemers, 2010). Recognizing species identity is required in many contexts, for example during mate recognition or predator avoidance. Anurans, for instance, employ acoustic signals intraspecifically for mate recognition (Ryan and Rand, 1993; Gerhardt and Huber, 2002), while vervet monkeys and meerkats distinguish visually between various (heterospecific) predators and react with predator-specific referential alarm calls (terrestrial, ground or aerial predator; Seyfarth et al., 1980; Manser, 2001). Furthermore, the recognition of heterospecifics can be ecologically advantageous if species share similar ecological requirements, e.g., in their diet, habitats or roosting requirements. Potential benefits include the formation of inter-specific foraging associations to improve feeding efficiency (Monkkonen et al., 1996), the eavesdropping on the activity of other individuals to gain information about available food (Übernickel et al., 2012) or shelter (Ruczynski et al., 2007).

Acoustic cues and signals play an important role for species recognition in many animals, including anurans, birds, insects and mammals (e.g., Ryan and Rand, 1993; Bradbury and Vehrencamp, 1998). Beyond species-specific information used for species recognition, acoustic cues and signals can carry several other information about the individual, for example about its morphology (e.g., large body size is related to low call frequency in frogs; Gerhardt and Huber, 2002), its behavior (e.g., foraging or not; Schnitzler and Kalko, 2001; Jones and Siemers, 2011) or certain external situations (e.g., presence of predator; Seyfarth et al., 1980; Manser, 2001). Acoustic stimuli thus provide a variety of information about an individual over some distance to other individuals in the vicinity.

**Figure 1:**
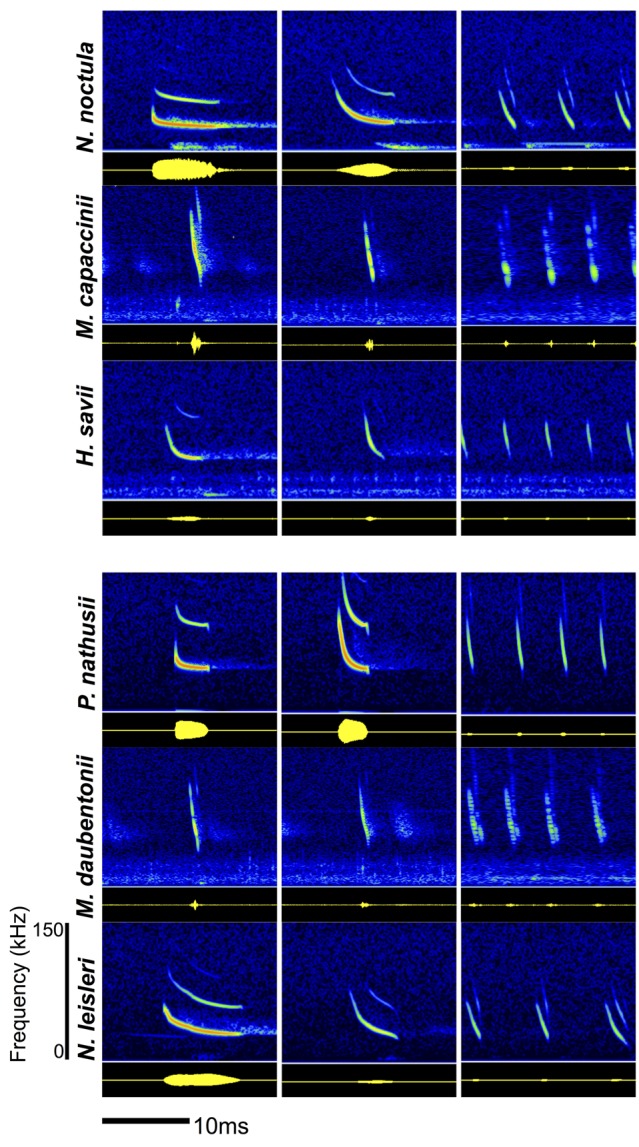
**Spectograms (top panel, amplitude color coded) and oscillograms (below, yellow on black) of the echolocation calls of all six recorded bat species.** The echolocation calls show species-specific differences and a characteristic change over the course of a feeding event (search phase, approach phase, feeding buzz).

Echolocating bats are particularly interesting for studying acoustic information transfer because they employ two different types of calls: social calls and echolocation calls. Social calls are used for social interactions between individuals (Barclay et al., 1979), while in contrast, ultrasonic echolocation calls are emitted by the bat for its own orientation, navigation and also for foraging in many species (Fenton, 1984; Schnitzler and Kalko, 2001; Neuweiler, 2003; Schnitzler et al., 2003). Echolocation calls are often species-specific, each species having a unique spectro-temporal structure (Barclay, 1999; Siemers et al., 2001; Obrist et al., 2004; Siemers and Schnitzler, 2004). In addition, this spectro-temporal structure is flexibly adapted to the habitat and behavioral task (Schnitzler and Kalko, 2001; Jones and Siemers, 2011). Particularly during foraging, the echolocation call sequence undergoes strong changes in its acoustic spectro-temporal structure (Kalko, 1995; Bradbury and Vehrencamp, 1998; Schnitzler and Kalko, 2001; Siemers, 2006). The search phase is characterized by calls emitted at a regular repetition rate. Upon prey detection, calls become shorter, more broadband and are emitted with an increasing repetition rate (approach phase) until the feeding buzz of up to 200 calls per second just before the capture (Siemers, 2006; Figure 1). Since echolocation calls belong to the loudest animal vocalizations (Holderied and von Helversen, 2003; Surlykke and Kalko, 2008), they are also audible to other bats, prey and predators over considerable distances of tens to a hundred or more meters, depending on species (Jones and Siemers, 2011). Echolocation calls are therefore an inevitably distributed source of information for other bats in the vicinity (Jones and Siemers, 2011), which may potentially eavesdrop on this available information about species identity and foraging activity.

The putative communicative function of echolocation calls has received considerable attention. Within their own species (intraspecifically), some species recognize sex (Kazial and Masters, 2004) and individual identity (Kazial et al., 2008; Yovel et al., 2009) of a conspecific based on echolocation calls and can show sex-specific behavioral responses in the field (Knörnschild et al., 2012). In a foraging context, playback experiments in the field showed that foraging bats approached conspecific feeding buzzes, probably using these signals as an indicator of food availability (Barclay, 1982; Fenton, 2003; Gillam, 2007; Dechmann et al., 2009). In contrast to intraspecific communication, interspecific communication, i.e., the communication between different species, has received little attention, particularly in the field. Two studies showed that bats in captivity are able to differentiate the echolocation calls of conspecifics from those of heterospecifics (Voigt-Heucke et al., 2010) and even differentiate between the echolocation calls of multiple heterospecifics (Schuchmann and Siemers, 2010). To the best of our knowledge, only one study to date has shown interspecific eavesdropping in wild and freely behaving bats, testing a species-rich neotropical bat community (Übernickel et al., 2012). The study tested two trawling bats *Noctilio leporinus* and *N. albiventris* that are sister species with similar echolocation call structure (yet differing in call frequency) and foraging ecologies. Both reacted to the buzz calls and, partially, to the search calls of the other. However, they did not react to any calls of *Saccopteryx bilineata*, a species with different call design and foraging ecology (open-space forager). In contrast, *S. bilineata* did not react to any calls of conspecifics or acoustically or ecologically similar heterospecifics. The results of Übernickel et al. (2012) suggest a relation between reaction strength and acoustic similarity that in turn is related to ecological similarity. Additionally, many other factors, including diet, prey density and distribution, typical foraging behavior, social structure or phylogeny, are likely to contribute, suggesting that reaction strength can vary strongly between different species (Ord and Stamps, 2009), requiring additional studies with different species.

Here, we investigated eavesdropping on the echolocation calls of bats within and across species boundaries. Using a Palearctic community of insectivorous bats, we tested for effects of species identity and call-type on the behavior of four different bat species during foraging. Our general hypothesis postulates that foraging bats evaluate the profitability of foraging patches based on the echo-acoustic information of other bats present in the hunting ground. Since profitable foraging patches can be indicated by foraging-specific calls (feeding buzzes) of species with similar foraging ecology, we predicted that the bats' reactions depend on the call-type and species identity of the calling species. We conducted the study in Germany and Bulgaria, testing in each country one bat species foraging in open-space (i.e., hunting prey in the open air) and one trawling bat species (i.e., taking prey from water surfaces). We presented playbacks of conspecific and different heterospecific species having the same and different foraging ecologies to test for the influence of call-type and foraging ecology. First, we predicted that bats would react more to feeding buzz echolocation calls than to search phase echolocation calls, as only the former indicate a potential food source. Second, we predicted that bats would react more strongly to the echolocation calls (both search calls and feeding buzzes) of conspecifics than to those of heterospecifics. Third, we predicted that bats would react more to the echolocation calls of heterospecific species with a similar feeding ecology than to heterospecifics with a dissimilar feeding ecology.

## Methods

### Study sites

We conducted fieldwork in Northern Bulgaria, within ca. 80 km around the village of Tabachka, and in South-East Germany, within ca. 35 km around the city of Munich. We selected a total of 16 sites on open meadows and next to lakes (i.e., areas used by bats as hunting grounds) for call recordings and playback experiments. The sites were covered by low vegetation such as grass, bushes and, in some cases, a few trees. We recorded echolocation calls at four sites (two lakes and two meadows) in Bulgaria and at five sites (four lakes and one meadow) in Germany. All playback experiments were conducted next to lakes at six sites in Bulgaria (including the two lake sites also used for call recordings) and at seven sites in Germany (including the four lake sites also used for call recordings). The distance between recording sites was minimally 23 km and maximally 100 km in Bulgaria and 8–59 km in Germany. For playback sites, distances were 30–123 km in Bulgaria and 7–57 km in Germany. Since none of the bats were marked individually, we cannot ensure that each recording was from a different individual or that each playback was presented to a different individual. However, at all sites we observed at least three and as many as six individuals per night. To avoid presenting individuals their own call recordings, we presented at each playback site only calls that had been recorded at a different site.

### Call recording

We recorded echolocation call sequences of six Vespertillionid bat species for subsequent playbacks (Figure 1): two open-space foragers and one trawling bat in each country. In Bulgaria, we recorded calls from *Nyctalus noctula* (open-space), *Hypsugo savii* (open-space) and *Myotis capaccinii* (trawling). In Germany, we recorded *Nyctalus leisleri* (open-space), *Pipistrellus nathusii* (open-space) and *Myotis daubentonii* (trawling; Figure 1). Recordings were conducted during 2 weeks of May 2011 in Germany and 2 weeks of June 2011 in Bulgaria during the first 2 h after sunset every evening for one night per recording site. We obtained an average of ca. 100 call sequences per night (and thus per recording site). We recorded the calls of foraging bats onto a ToughBook Laptop (Panasonic, New Jersey, USA) using an ultrasonic microphone (CM16/CMPA, Avisoft, Berlin, Germany) connected to an USG 116 Hm soundcard (Avisoft) and the software RECORDER USGH v. 3.4 (Avisoft) at 250 kHz sampling frequency and 16 bit resolution. The microphone was vertically mounted on a tripod 35 cm above ground level. Recordings were triggered manually when a bat was visually detected and consisted of 3 s before and after triggering.

### Call analysis and playback preparation

Recorded species were identified during call recording by observation with night vision goggles (ATN PVS7-3, ATN, San Francisco, USA; based on body size and foraging style) and afterwards in Selena software (Animal Physiology, University of Tübingen, Germany; FFT 256, frequency resolution 125 Hz and auto padding) based on call shape and frequency of the spectrogram. We excluded recordings if visual observation and call analysis did not match.

In total, across all six recorded bat species, we obtained a total of 1478 recordings of 6 s duration. For the playbacks, we selected 1-s segments with a good signal-to-noise ratio containing either only search phase calls or only feeding buzzes. The number of selected segments differed between playback species and call types (search calls and feeding buzzes) and mostly ranged from 11–32 segments, except for *H. savii* (2 feeding buzz segments) and *N. noctula* (70 search call segments). We created final playback files of 10 s duration by replicating each segment of 1 s duration. Final playback files were high-pass filtered at 15 kHz and normalized to −3 dB full scale of the playback system. As control stimuli we used ten different pure-tones of 10 s duration ranging from 20 kHz to 65 kHz in 5 kHz steps. Altogether, we had seven different playback types (six test playbacks, i.e., two call types from three species, and one control). All playbacks were conducted at 250 kHz sampling frequency and 16 bit resolution. In Bulgaria, we randomly selected each night five files with search calls and five files with feeding buzzes from each of the three recorded species. Together with the ten control files, this yielded 40 playback files per night. In Germany, we presented 30 playbacks per night by randomly selecting ten files with search calls (out of the 150 files of all three species), ten files with feeding buzzes (out of 150 files of all three species) and ten control stimuli. For each playback session, the selected files (40 in Bulgaria, 30 in Germany) were presented in random order.

### Playback experiments

We conducted playback experiments during May and July in Germany and June in Bulgaria at the foraging sites of four Vespertillionid bat species (one open-space and one trawling species in each country). In Bulgaria, the focal species were *Nyctalus noctula* (open-space forager) and *Myotis capaccinii* (trawling bat). In Germany, the focal species were *Pipistrellus nathusii* (open-space forager) and *Myotis daubentonii* (trawling bat). We presented three types of call recordings to each focal species, namely calls of conspecifics (i.e., belonging to the same species) and of two different heterospecifics. The two heterospecific species differed in their foraging ecology (one open-space or trawling forager). Therefore, each focal species had playbacks from conspecifics, from one heterospecific species with the same and one with a different foraging ecology. Playbacks were presented with an ultrasonic loudspeaker (ScanSpeak; Avisoft) and an USG Player 116 soundcard (Avisoft). The loudspeaker had an overall low-pass characteristics of −12 dB between 10 and 110 kHz and a maximum output level of 100 dB SPL (re. 20 μPa) at 1 m distance and was located 1 m from the lakeshore and 50 cm above the ground pointing toward the lake. We positioned a microphone (details see above) next to the speaker to record the focal bats for posterior identification. Additionally, we used a bat detector (100D Petterson, set to heterodyne) and night vision goggles (ATN PVS7-3, San Francisco, USA) to follow the behavior of the focal bat.

We defined the experimental area as a circle with a radius of ten meters around the loudspeaker and used bushes and trees as reference points for distance estimation. Whenever a bat entered the experimental area, a randomly and blindly chosen playback file was presented. Simultaneously, we recorded the echolocation calls of the focal bat and observed its flight behavior visually. The bats behavior was scored during the experiment in the field. When the bat changed its flight direction toward the loudspeaker, we scored this as a “reaction” to the playback. Otherwise, when the bat did not change its flight direction, this was scored as “no reaction.” We only observed one trial where a bat turned away from the loudspeaker, which was excluded. Trials in which a bat was initially flying directly toward the loudspeaker were excluded because a potential reaction could be due to the loudspeaker being a physical obstacle. The species of the focal individual was identified during the experiments visually (night vision goggles; ATN PVS7-3) based on body size and foraging style (i.e., in open space or trawling) and afterwards based on spectrograms of the recorded echolocation calls. Trials were excluded if visual observation and call analysis did not match and if the focal bat did not belong to our focal species (*Nyctalus noctula* and *Myotis capaccinii* in Bulgaria, *Pipistrellus nathusii* and *Myotis daubentonii* in Germany).

### Data analysis

For each focal species, we counted the number of bat passes showing a “reaction” or “no reaction” to each playback type. Statistical analyses were conducted in R 2.11.0 (R Development Core Team, 2008). Per focal species, we computed two generalized linear models (GLM) for binomial data to test for differences in the number of reacting bat passes. First excluding the reactions to the control stimuli, we calculated GLMs with playback species (three levels) and call type (two levels) as fixed factors to test for species- and call type-specific reactions. For the second GLM, we included the reactions to the control stimuli and used playback type (combining bat species and call type) as a single fixed factor with seven levels to test for further differences between call types. Pair-wise comparisons between factor levels were performed with the *multcomp* package with single-step adjusted *p*-values.

## Results

### Overall reaction to call playbacks

**Figure 2:**
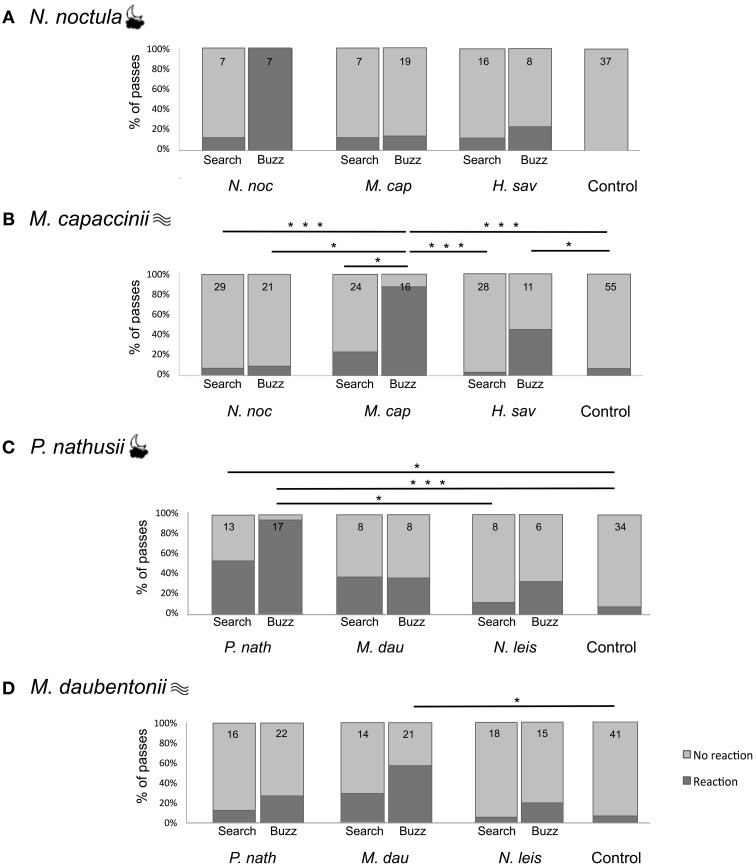
**Percentage of bat passes showing a reaction (dark gray) and no reaction (light gray) in response to the playback of echolocation calls of different species. (A)** Reactions of *N. noctula* and **(B)** *M. capaccinii* in Bulgaria. **(C)** Reactions of *M. daubentonii* and **(D)** *P. nathusii* in Germany. The focal species is indicated at the top left of each panel, with symbols indicating its foraging style [cloud (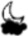) = open-space forager; waves (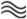) = trawling forager]. The playback species is indicated below the bar plots. The small numbers in each bar are the number of recorded passes. Lines and asterisks indicate significant differences between playback types (^*^0.05 > *p* ≥ 0.01; ^**^0.01 > *p* ≥ 0.001; ^***^p < 0.001) based on multiple comparisons with Tukey contrasts between all seven different playback types.

We presented four focal bat species the search echolocation calls and feeding buzzes of three con- and heterospecific bat species as well as sinusoidal control stimuli. We counted the number of bat passes showing a reaction to the playback, defined as a change of flight direction toward the loudspeaker. All four focal species reacted in less than 9% of the trials to the control stimuli (Figure 2). In contrast, there was a large variation in the response of different focal species to the different playbacks; bats reacted in 4–53% of the trials to search calls and in 10–100% of the trials to feeding buzzes. The minimal adequate GLM for *Nyctalus noctula* (Figure 2A) as focal species included both fixed factors playback species and call type, and the interaction between both factors. For the other three focal species (*Myotis capaccinii*, *Pipistrellus nathusii*, *Myotis daubentonii*; Figures 2B–D), the minimal adequate GLM included the fixed factors playback species and call type, but not their interaction.

### Reaction to different call types (search calls and feeding buzzes)

Call type was included in the minimal adequate GLM of all four focal species; thus, call type influenced the number of reacting bat passes. For each focal species, we conducted *post-hoc* multiple comparisons between the overall reactions to different call types (including all playback species). Although call type was included in the minimal adequate GLM of *N. noctula*, its overall reaction did not differ between search and buzz calls (adj. *p* = 0.446, *post-hoc* tests with manual contrasts to account for factor interaction). The three other focal species (*M. cappaccinii*, *P. nathusii*, *M. daubentonii*) reacted more strongly to buzz calls than to search calls (Tukey *post-hoc* tests, adj. *p* = 0.034–<0.001).

### Reaction to different playback species

Playback species was included in the minimal adequate GLM of all four focal species; thus, the playback species influenced the number of reacting bat passes. For each focal species, we conducted *post-hoc* multiple comparisons between the overall reaction to different playback species (including search calls and feeding buzzes). The overall reaction of *N. noctula* did not differ between playback species (adj. *p* = 0.821–1.000), despite playback species being included in the minimal adequate model. The three other focal species (*M. cappaccinii*, *P. nathusii*, *M. daubentonii*) reacted overall stronger to conspecific playbacks than to heterospecific playbacks (Tukey *post-hoc* tests, adj. *p* = 0.03344–<0.001). In contrast, their reaction did not differ between the heterospecific species (Tukey *post-hoc* tests, adj. *p* = 0.2368–0.8433).

### Intra- and interspecific reaction to specific playback types

*Nyctalus noctula* (Figure 2A) reacted strongly to conspecific feeding buzzes (100%, *N* = 7; Figure 2A). However, it reacted rarely to conspecific search calls or to any heterospecific call type (13–25%) and it did not react at all to the control (0%). Nevertheless, none of these differences were significant (Figure 2A, Tukey *post-hoc* test, adj. *p* = 0.983–1). Due to this pattern, the minimal adequate model included both factors and their interaction, while the *post-hoc* tests showed that *N. noctula* does not generally react differently to any playback species or call type. *Myotis capaccinii* (Figure 2B) also reacted most strongly to conspecific feeding buzzes (88%, *N* = 16), and reacted equally strongly to buzzes of the heterospecific *H. savii* (45%, *N* = 11; Tukey *post-hoc* test, adj. *p* = 0.287). Both differed from the reaction to the control stimuli (7%, *N* = 55; adj. *p* = 0.0453–<0.001). Furthermore, the reaction of *M. capaccinii* to conspecific feeding buzzes was also stronger compared to conspecific search calls (28%, *N* = 25; adj. *p* = 0.016), which did not differ from the control stimuli (7%, *N* = 55; adj. *p* = 0.216). Likewise, *M. capaccinii* did not react to any calls of *N. noctula* (7%, *N* = 29; 9%, *N* = 21), which did not differ from the reaction to the control stimuli (adj. *p* = 1.000), but differed from the reaction to conspecific feeding buzzes (adj. *p* = 0.001–<0.001). *Pipistrellus nathusii* (Figure 2C) reacted most strongly to conspecific feeding buzzes (94%, *N* = 17) and equally strongly to conspecific search calls (54%, *N* = 13; adj. *p* = 0.268). Both reactions differed from the reactions to the control stimuli (9%, *N* = 34, adj. *p* = 0.0375–<0.001). The reactions to the calls of *M. daubentonii* (38%, *N* = 8) and the feeding buzzes of *N. leisleri* (33%, *N* = 6) were intermediate between the reactions to conspecific calls and the control, but not significantly different to either of them due to the small sample size. *Myotis daubentonii* (Figure 2D) reacted, like the other species, most strongly to conspecific feeding buzzes (55%, *N* = 22), which differed significantly from the control stimuli (7%, *N* = 41, adj. *p* = 0.00397). The remaining reactions to conspecific search calls and heterospecific calls were intermediate between the conspecific feeding buzzes and the control stimuli, without any significant differences.

## Discussion

Generally, focal species reacted more strongly to playbacks of echolocation calls than to playbacks of control stimuli, more strongly to feeding buzzes than to the search calls, and more strongly to the calls of conspecifics than to those of heterospecifics. The detailed reaction patterns differed between focal species, with some species potentially showing signs of heterospecific eavesdropping.

### Reaction to different call types (search calls and feeding buzzes)

Bats only emit feeding buzzes just before attacking prey (Kalko, 1995; Bradbury and Vehrencamp, 1998; Schnitzler and Kalko, 2001; Siemers, 2006). Consequently, feeding buzzes provide information on prey availability and the profitability of a foraging patch to bats in the vicinity. The use of this cue potentially increases the chance of the eavesdropper to find food (Barclay, 1982; Fenton, 2003; Gillam, 2007; Dechmann et al., 2009). This information is not present in search phase calls. We thus predicted a call type-specific reaction, which was supported in three species (*M. capaccinii*, *P. nathusii* and *M. daubentonii*) by an overall stronger attraction to feeding buzzes compared to search calls.

The detailed analysis per playback type supported this finding on the intraspecific level for one species. *M. capaccinii* reacted significantly stronger to feeding buzzes than to search phase calls. The data of the other three species (*N. noctula, P. nathusii* and *M. daubentonii*) also showed stronger reactions to feeding buzzes than to search calls. However, these differences were not significant, but still showed different patterns between species. *N. noctula* and *P. nathusii* reacted almost always to conspecific feeding buzzes, but *M. daubentonii* only to about half of the playbacks. *N. noctula* and *M. daubentonii* reacted rarely to search calls while *P. nathusii* reacted to about half of the playbacks. These results suggest an attraction of bats to the feeding buzzes of other individuals from the same species, supporting previews findings about bats using conspecific buzzes as an indicator for food availability (Barclay, 1982; Fenton, 2003; Gillam, 2007; Dechmann et al., 2009).

On an interspecific level, we also found evidence of eavesdropping on the feeding buzzes of heterospecifics in one species. *M. capaccinii* reacted equally to the feeding buzzes of conspecifics and those of *H. savii*. Both reactions differed significantly from the control and other playbacks. The other three focal species reacted sometimes more to heterospecific feeding buzzes compared to search calls, though never strongly and significantly.

### Reaction to different playback species

Three species (*M. capaccinii*, *P. nathusii* and *M. daubentonii*) reacted more strongly to conspecific than to heterospecific echolocation calls, supporting our prediction of species-specific reactions. The overall reaction to different heterospecifics, however, did not differ for any of the focal species. Likewise, none of the focal species reacted overall similarly to calls of conspecifics and of heterospecific with similar foraging ecology. Our prediction of foraging ecology-dependent reaction was thus not confirmed for all calls of a species.

### Intra- and interspecific reaction to specific playback types

We found no general and unequivocal evidence for interspecific eavesdropping. While the lack of reaction to ecologically dissimilar species is in line with our prediction, it is not supported by a matching reaction to ecologically similar heterospecifics. For example, the open-space foragers *N. noctula* and *P. nathusii* reacted only little to the playbacks of the heterospecific trawling bats *M. capaccinii* and *M. daubentonii*. However, both species also did not react to playbacks of heterospecific open-space foragers (*H. savii* and *N. leisleri*), indicating that they might not react at all to any heterospecific. Only *M. capaccinii* showed a clear attraction to heterospecific echolocation calls, namely to the feeding buzzes of *H. savii*, despite these species' overall difference in foraging habitats (trawling and open-space foragers, respectively). However, *M. capaccinii* does not only forage above water surfaces but also in open airspace (Dietz et al., 2009), which is the typical hunting habitat of *H. savii* (Dietz et al., 2009). *M. capacinii* might thus have reacted to the feeding buzzes of a heterospecific with partially overlapping foraging ecology, which indicated a profitable aerial foraging spot. In contrast to Übernickel et al. (2012), this raises the possibility of interspecific eavesdropping across foraging guilds. However, another possibility is that the reaction of *M. capaccinii* is due to the acoustic similarity of the echolocation calls, particularly the feeding buzzes, of *M. capaccinii* and *H. savii* (Balcombe and Fenton, 1988; Übernickel et al., 2012). To test this, it would be interesting to see if *M. capaccinii* reacts even more strongly to the trawling bat *M. daubentonii*, which is also acoustically similar, yet overlaps more in foraging ecology than *H. savii*.

### Eavesdropping in bat communities

The occurrence and potential benefits of eavesdropping will be determined by multiple factors, including a species' foraging style and social system, the species similarity with sympatric species, and the conditions of its habitat, such as prey availability (Dechmann et al., 2009; Jones and Siemers, 2011; Übernickel et al., 2012). Eavesdropping enables bats to extend their perception beyond the limited detection range of their own echolocation system and to gain information about prey availability, profitable foraging patches, roosting sites and the behavior of other individuals (e.g., Barclay, 1982; Gillam, 2007; Ruczynski et al., 2007; Dechmann et al., 2009). On the other hand, eavesdropping might constitute a cost for the bat that is being eavesdropped upon, potentially leading to competition between interacting individuals. The costs and benefits in a foraging context are determined by the availability of resources. For instance, females of the bat *Noctilio albiventris* eavesdrop on conspecific calls to detect large, but patchily distributed insect swarms (Dechmann et al., 2009). Since the swarms are so large that they cannot be monopolized and exploited by a single individual, eavesdropping does not incur any costs and has the benefit of an increased detection range of the swarms. In contrast, for bats that feed on more distributed prey items, eavesdropping will be costly for the bat that is eavesdropped upon, particularly in times of scarcity and high energy demand, and potentially lead to resource defence (Barlow and Jones, 1997).

We predicted that bat species would react more strongly to ecologically similar species, i.e., species with similar foraging habitats, foraging styles and prey spectra. Such ecological similarity is also reflected in morphological and echo-acoustic similarity between species, which influences their maneuverability, flight speed, bite force, hunting style and prey perception ability (Balcombe and Fenton, 1988; Swartz et al., 2003; Siemers and Schnitzler, 2004). As a consequence, ecologically dissimilar species regularly differ in additional aspects such as their body size, flight speed, foraging style and call shape and frequency, which are all potential explanations for low reaction to playbacks of ecologically dissimilar species. For example, *N. noctula* forages high up in the air and might thus not be attracted to the calls of the smaller species hunting closer to water bodies and background structures (*M. capaccinii*, *H. savii*). The low proportion of reactions from medium (*M. capaccinii*, *M. daubentonii*) and small sized bat species (*P. nathusii*) to the playbacks of the bigger bats (*N. noctula* and *N. leisleri*) can be due to marked body size differences, which again correlate with differences in maneuverability, flight speed, bite force and prey spectrum. Balcombe and Fenton (1988) suggested that bats react most to calls that are acoustically similar to their own calls, based on the idea that acoustic similarity reflects ecological similarity. This idea is confirmed by the attraction of *M. cappaccinii* to the feeding buzzes of *H. savii*, which have feeding buzz calls that are similar both in frequency and repetition rate (Figure 1). However, we did not find a reaction to the playback of heterospecific echolocation calls in other species pairs with a similar amount of acoustic similarity in the feeding buzzes (e.g., *N. noctula* and *M. cappaccinii* or *M. daubentonii* and *N. leisleri*). The species-specificity of echolocation calls is more pronounced for search calls than for feeding buzzes, which allows the possibility that bats were not able to tell species identity based on feeding buzzes alone. Further studies separating the effects of ecological and acoustic similarity would thus be interesting.

### Conflict of interest statement

The authors declare that the research was conducted in the absence of any commercial or financial relationships that could be construed as a potential conflict of interest.
